# Optical coherence tomography for the detection of electrothermal ureteral injury in an *ex-vivo* porcine model

**DOI:** 10.1117/1.BIOS.1.1.015001

**Published:** 2024-05-20

**Authors:** Dilara J. Long, Photini F. Rice, Joshua Routh, Sunchin Kim, Lucas Struycken, Julia Fisher, David Besselsen, Dominique Galvez, Ryan Walton, Jennifer K. Barton, John M. Heusinkveld

**Affiliations:** a The University of Arizona, Department of Biomedical Engineering, Tucson, Arizona, United States; b Midwestern University, Department of Pathology, Glendale, Arizona, United States; c The University of Arizona, Department of Urology, Tucson, Arizona, United States; d The University of Arizona, Department of Medical Imaging, Tucson, Arizona, United States; e The University of Arizona, Bioscience Research Laboratories, Statistics Consulting Laboratory, Tucson, Arizona, United States; f The University of Arizona, University Animal Care, Tucson, Arizona, United States; g The University of Arizona, James C. Wyant College of Optical Sciences, Tucson, Arizona, United States; h The University of Arizona, Department of Obstetrics and Gynecology, Tucson, Arizona, United States

**Keywords:** optical coherence tomography, endourology, ureter, thermal ureteral injury, iatrogenic ureteral injury, minimally invasive imaging

## Abstract

**Significance:**

The current technology has limited ability to detect lateral thermal spread of injury caused by electrosurgical devices during gynecologic procedures.

**Aim:**

We aim to assess the feasibility of endoscopic optical coherence tomography (OCT) to detect electrothermal ureteral damage.

**Approach:**

Electrothermal energy was externally applied to nine explanted porcine ureters. Three segments of each ureter were treated for 5 s at low (16 W), medium (26 W), and high (36 W) powers (n=27 segments). Volumetric OCT images were acquired using a swept source OCT laser endomicroscopy system. OCT datasets were visually inspected for characterization of normal and electrothermally injured tissue architecture. Ground-truth comparisons were made with histology to validate the presence of lesions and to compare lesion size measurements using Pearson’s correlation coefficient. Three physicians were trained to identify OCT images of normal and injured ureters. Physician lesion detection accuracy was tested with 126 OCT images (63 normal and 63 injured). The effect of treatment power on lesion length as measured with OCT was compared using a one-way analysis of variance.

**Results:**

Transmural electrothermal injury was identified on OCT images for all but one histology-confirmed lesion (22/23, 95.7%). The average sensitivity and specificity for physician lesion detection were 82% and 96%, respectively. The mean lesion size measured on OCT was 3.6±1.9, 4.4±1.3, and 7.0±2.9  mm for low, medium, and high powers, respectively (p=0.024). A comparison of lesion size measured on OCT and histology revealed a moderate positive correlation (r=0.65, p=0.00087).

**Conclusions:**

Endoscopic OCT could fulfill the unmet clinical need for the timely detection of electrothermal ureteral injury.

Statement of Discovery We describe the first-known feasibility study of endoscopic optical coherence tomography to detect unintentional ureteral damage caused by electrosurgical devices during gynecologic procedures.

## 1 Introduction

Iatrogenic ureteral injury is a rare (<1%) yet serious complication of pelvic and abdominal surgery.^1^^–^^3^ The ureters are particularly susceptible to lateral spread of electrothermal injury due to their proximity and resemblance to anatomic structures undergoing intentional electrocoagulation, especially during laparoscopic hysterectomies.^1^ Injury is frequently unrecognized until the sequelae of ischemia, scar and stricture formation, fistulas, and/or adhesions present days, weeks, or months postoperatively.^4^^–^^6^

There is an unmet clinical need for a standardized approach that enables timely and direct assessment of ureteral integrity in the operating room. The current intraoperative surveillance methods, including direct visualization of gross injury or indirect visualization of urine extravasation with cystoscopy, have detection rates of as low as 35% and 53%, respectively,^1^ and are more specific for other types of injuries such as kinking and laceration.^6^^–^^9^ Earlier detection is necessary to reduce morbidity, increase ease of repair, and improve patient outcomes.^10^^,^^11^ A method to diagnose, localize, and define the severity of thermal injury could also facilitate targeted management and repair.^12^

Microscopy can reveal early signs of coagulative injury not visible to the naked eye but requires tissue resection.^13^^–^^15^ Electrocoagulation confers changes in tissue optical properties, which can be detected using non-destructive high-resolution imaging techniques such as optical coherence tomography (OCT).^16^^–^^18^ OCT is analogous to ultrasound but uses near-infrared light to provide histology-like images up to 3 mm in depth. OCT can be implemented as a sub-millimeter endoscope to provide cross-sectional depth visualization of microstructural features in luminal organs, such as the ureters.^19^^–^^22^ The objective of this study was to assess the feasibility of OCT endoscopy as an approach to detect electrothermal injury in an *ex-vivo* porcine ureter model.

## 2 Methodology

### 2.1 Ureter Preparation and Application of Electrothermal Energy

This study was conducted according to an *ex-vivo* imaging protocol approved by the Institutional Animal Care and Use Committee at the University of Arizona. Twelve ureters were explanted from six female domestic Yorkshire swine within 2 h of euthanasia. Tissues were stored and flushed in 0.9% saline to prevent drying. Two ureters were set aside as untreated controls. Of the 10 remaining ureters, one was used to test optimal energy parameters, and nine were treated by the following protocol. A conventional bipolar high-frequency electrosurgical generator (Model 26021, Karl Storz, Tuttlingen, Germany) and a bipolar cautery tip (part no. 67-1065, Gemini Cautery System, Gaithersburg, Maryland, United States) were used for the external application of electrothermal energy. A weighted lever was used to elicit a controlled contact pressure of 105 g over $∼4\;{\mathrm{mm}}^{2}$ of the external ureteral surface.

**Fig. 1:**
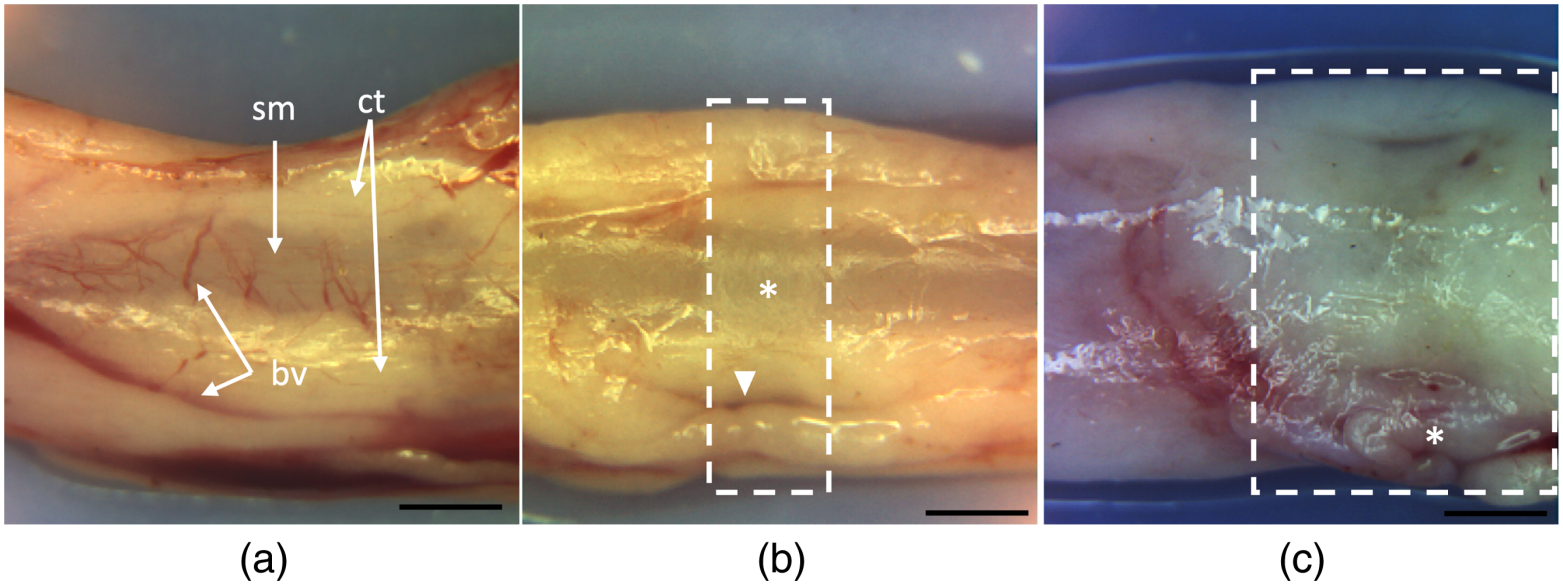
Treatment endpoints as determined by external visual inspection following 5 s of treatment. (a) Porcine ureter segment treated with 16 W of energy (low power), characterized by minimal to no grossly visible signs of injury. Normal appearing external connective tissue (ct), underlying smooth muscle (sm), and bright red blood vessels (bv) are appreciated. (b) Medium-power lesion treated with 26 W of energy (white box), characterized by subtle coagulative injury with tissue whitening, smooth muscle contraction (asterisk), and dark, coagulated blood vessels (arrowhead). (c) High-power lesion applied with 36 W (white box), characterized by obvious signs of coagulation (whitening) and, in some cases, gross ablation with darkening and charring of tissue. Scale bar=2  mm.

Three treatment endpoints were selected based on externally visible signs of injury with 5 s of energy application (Fig. 1). Low-power lesions had mild to absent visible signs of injury. Medium-power lesions showed subtle coagulative injury (whitening and dehydration). High-power lesions had obvious signs of injury, including charring, contraction, desiccation, or evaporation. Three segments of each ureter were treated at low (16 W), medium (26 W), and high (36 W) powers with ∼3  cm between regions (n=27 segments). The order of power treatments was randomized among ureters.

### 2.2 OCT Image Acquisition, Processing, and Analysis

Immediately following treatment, ureters were mounted onto a dedicated 7F low-profile optical probe connected to a swept-source OCT volumetric laser endomicroscopy imaging system (95501-LP, Ninepoint NVisionVLE), described previously.^23^ In short, this technology uses a near-infrared swept source laser with a wavelength range from 1260 to 1360 nm and a 50-kHz repetition rate for the continuous acquisition of two-dimensional (2D) cross-sectional OCT images. The axial and lateral resolutions of the system were 7 and 40  μm, respectively. An automatic spiral-scanning pullback mechanism was used to acquire a series of up to 1200 stacked 2D images (6 cm) in ∼90  s. Ureters were imaged in their entirety to include both normal and treated areas.

Image processing and analysis were conducted using ImageJ (National Institutes of Health, Bethesda, Maryland, United States) by an experienced observer. In the grayscale images, darker areas corresponded to higher signal reflectivity. The same observer qualitatively compared OCT images and the corresponding histology to characterize features of normal and injured ureters. Following confirmation of injury on histology, definitive criteria for injury were developed and used to label OCT images as normal or injured. The lesion size (mm) was determined by multiplying the number of consecutive slices with the injury by an image slice thickness of 50  μm. Given the observer’s prior exposure to histology and adjacent images in OCT volumes, image labeling was considered unblinded. The extent of ureteral wall involvement and the severity of damage were assessed and compared among power groups.

### 2.3 Histology Processing and Analysis

The proximal, middle, and distal segments of two untreated control ureters (n=6 segments) and segments of injured areas with adjacent untreated tissue (n=27 segments) were processed for histology using routine protocols. Longitudinal sections of 6-μm thickness were acquired at 125-μm intervals. Hematoxylin and eosin (H&E) and Masson’s trichrome-stained slides were analyzed using a brightfield microscope (BX41, Olympus, Japan). Slides with the maximal lesion size were formally reviewed and measured by a board-certified pathologist.

### 2.4 Physician Detection Study

Three board-certified physicians specializing in Interventional Radiology, Pathology, and Urology, with no prior OCT experience, were trained to differentiate normal and injured ureters using 14 example OCT images. Physician detection accuracy was evaluated using a dataset of 126 OCT images with equal representation of normal (n=63 images) and injured (n=63 images) areas. The injured image dataset encompassed all lesions with confirmed injury on ground-truth histology. Based on the labeled lesion start and end slice number, three images of each lesion were acquired at 12.5%, 50%, and 75% longitudinal intervals. Images with significant distortion or unique artifacts, which were not discussed in training were excluded from the test dataset. For each ureter imaged, four to six OCT images of normal (untreated) areas were selected to match the number of injured images. We assured a minimum of 0.5-cm (100 images) distance between images and from injured areas.

### 2.5 Statistical Analysis

The average lesion size (mm) for samples that met the criteria for injury on both OCT and histology was compared among power groups, presented as mean±standard deviation for low (n=5 segments), medium (n=8 segments), and high (n=9 segments) power groups. Pearson’s correlation coefficient was used to assess the linear relationship among measurements of lesion size on OCT (n=27 segments) and histology (n=27 segments), including false positives and false negative measurements. A one-way analysis of variance (ANOVA) with post-hoc Tukey’s honest significant difference (HSD) test was used to compare lesion size as measured with OCT among power groups. Lesions identified on OCT that did not meet the criteria for injury on ground-truth histology were considered false positives, whereas lesions identified on histology that did not meet the criteria for injury on OCT were considered false negatives (see Sec. 3.2 for a description of the criteria). p-Values less than 0.05 were considered statistically significant. Statistical analysis and figures were generated using Rstudio (Version 2023.03.1+446).

## 3 Results

### 3.1 Comparison of Untreated Ureters on OCT and Histology

**Fig. 2:**
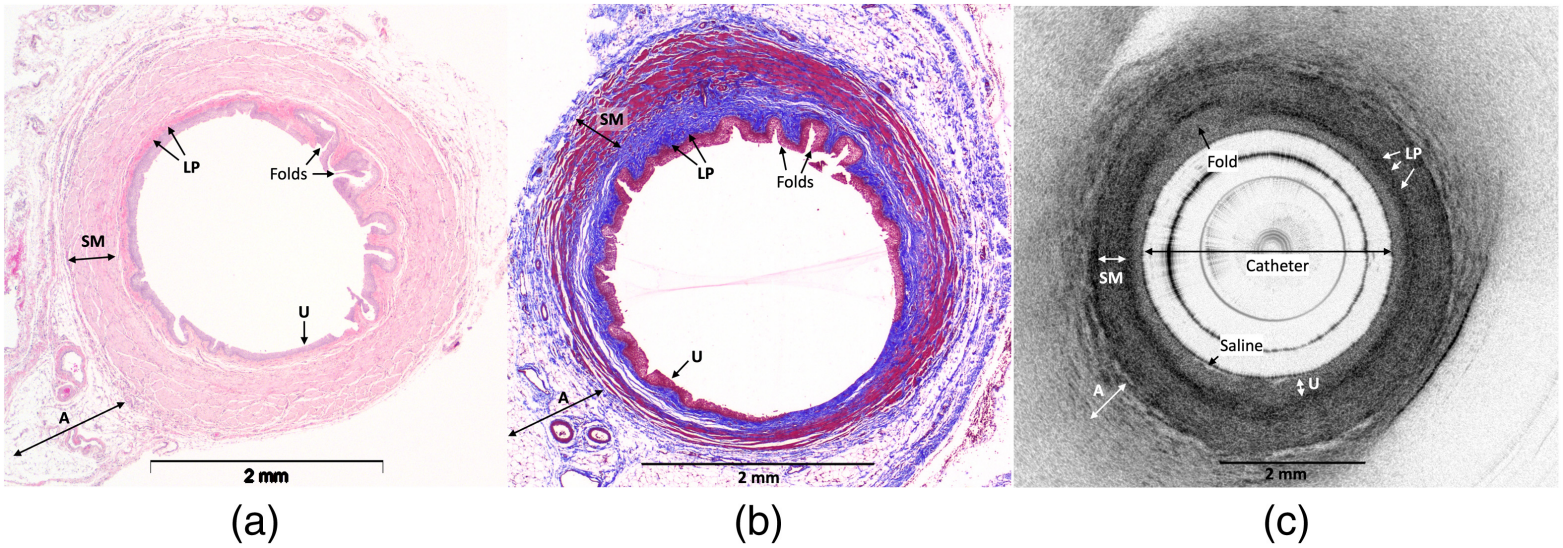
Comparison of untreated control porcine ureters on OCT and histology. (a), (b) Axial representations of an H&E and trichrome-stained ureter, respectively. (c) Example of an untreated porcine ureter on OCT images. Darker grayscale areas correspond to increased signal reflectivity and backscattering, which was more prominent in areas with higher collagen composition as shown in panel (b) (blue). U, urothelium; LP, lamina propria; SM, smooth muscle; A, adventitia.

Untreated areas on OCT images demonstrated spatial changes in contrast and reflectivity that corresponded with the normal histological features of the urothelium, lamina propria, smooth muscle, and adventitia (Fig. 2). The multilayered transitional cell urothelium was identifiable on histology in all samples. Regions of isolated epithelial denudation <10  mm in length were identified in ∼10% of untreated controls and in normal areas distant from treated areas. On OCT images, the urothelium was only within the visible resolution in larger, relaxed ureters, where it appeared as an inner hyporeflective (light) band in regions with mucosal folds. The underlying lamina propria, characterized as a well-organized layer of dense, fibrillary collagens on histology, consistently appeared as a thin, uniform, hyperreflective (dark) band on OCT images. The smooth muscle layer, consisting of alternating circular and longitudinal muscular fibers on histology, correlated with the large heterogeneous layer with a striated texture on OCT images. The adventitia was identified as the outer hyporeflective layer with a diffuse hyperreflective speckled pattern.

OCT image analysis was not possible in areas with two types of artifacts. First, areas with folds showed increased scattering and signal attenuation across all layers beyond the urothelium. Second, probe slippage or non-concentric misalignment during acquisition could cause severe apparent distortion in the imaged tissue.

### 3.2 Comparison of Injured Ureters on OCT and Histology

**Fig. 3:**
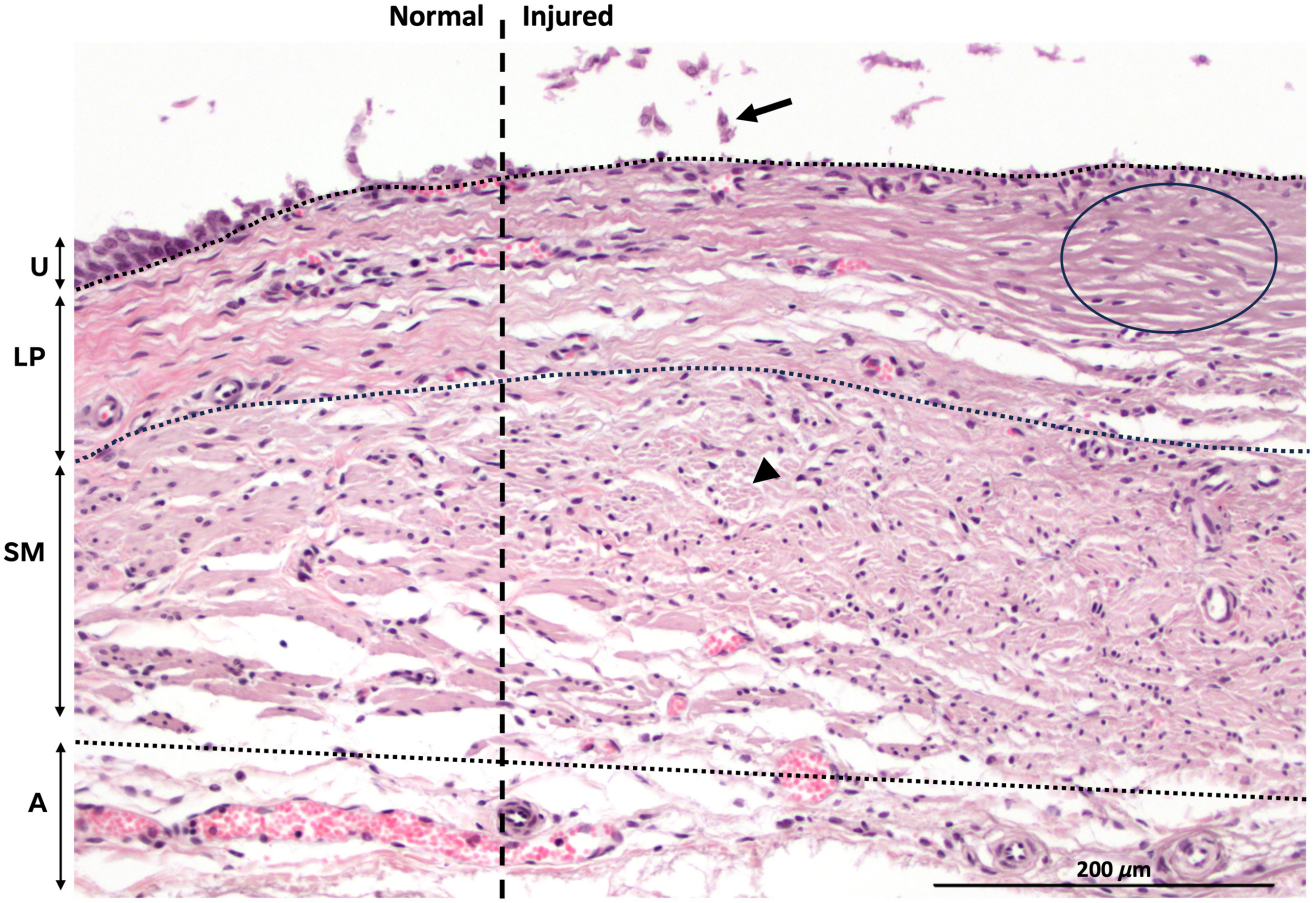
Characterization of electrothermal injury on histology (20× magnification). The dashed vertical line indicates an approximate boundary between normal (left) and injured (right) areas. The horizontal dotted lines represent the approximate boundary among tissue layers. Electrothermal injury was identified in areas with denuded uroepithelium (U) and sloughing of epithelial cells (arrow); coagulative denaturation of collagen fibers in the lamina propria (LP), which is best appreciated at the lesion center (circle); and fragmentation and swelling of smooth muscle (SM) fibers (arrowhead). The adventitia (A) with blood vessels is appreciated but was not used for the evaluation of injury.

**Fig. 4:**
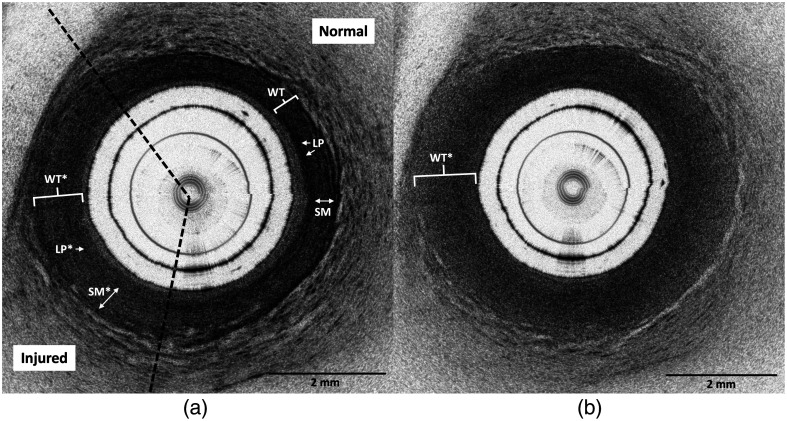
Comparison of the morphological appearance of an injured porcine ureter (a) near the boundary and (b) at the center of a lesion treated with high-power (36-W) electrothermal energy. (a) Compared with normal areas, injured areas showed lightening of the lamina propria (LP*), decreased contrast and heterogeneity in the smooth muscle (SM*), and increased wall thickness (WT*). The dotted lines show the approximate boundaries of the lesion, which fulfills both injury criteria. (b) Toward the lesion center, the circumferential extent of injury and the wall thickness (WT*) increase, and the tissue appears more homogeneous. Characteristic features of normal tissue are faint to absent.

Electrothermal injury was identified in 23/27 (85%) treated segments on ground-truth histology. Four areas treated at low power (16 W) did not meet histological criteria for injury. On histology, injury was characterized by a focal transmural lesion with coagulative denaturation of collagen bundles and swelling and fragmentation of smooth muscle fibers (Fig. 3). On OCT images, electrothermally injured areas were identified by signal homogenization and attenuation, which corresponded to decreased signal contrast and reflectivity, respectively (Fig. 4). An image stack that demonstrates one high-power (36-W) lesion among normal areas of tissue is provided in Video 1 (see Appendix). In general, tissue swelling, or an increase in wall thickness compared with adjacent normal areas, was appreciated in injured areas. However, swelling was excluded from formal injury criteria due to the variability in ureter sizes across animals and among proximal, middle, and distal ureteral segments.

Two consistent features were observed on OCT images and were used as criteria for injury. Criteria 1 was characterized by a reduction in signal reflectivity in the lamina propria layer, resulting in visible lightening of the inner dark band. Criteria 2 was defined as the loss of microstructural detail (i.e., decreased contrast and loss of heterogeneous striations) in the smooth muscle layer. These criteria were exclusively assessed in artifact-free regions and had to demonstrate continuity for at least 1/8 (45 deg) of the total circumference.

**Fig. 5:**
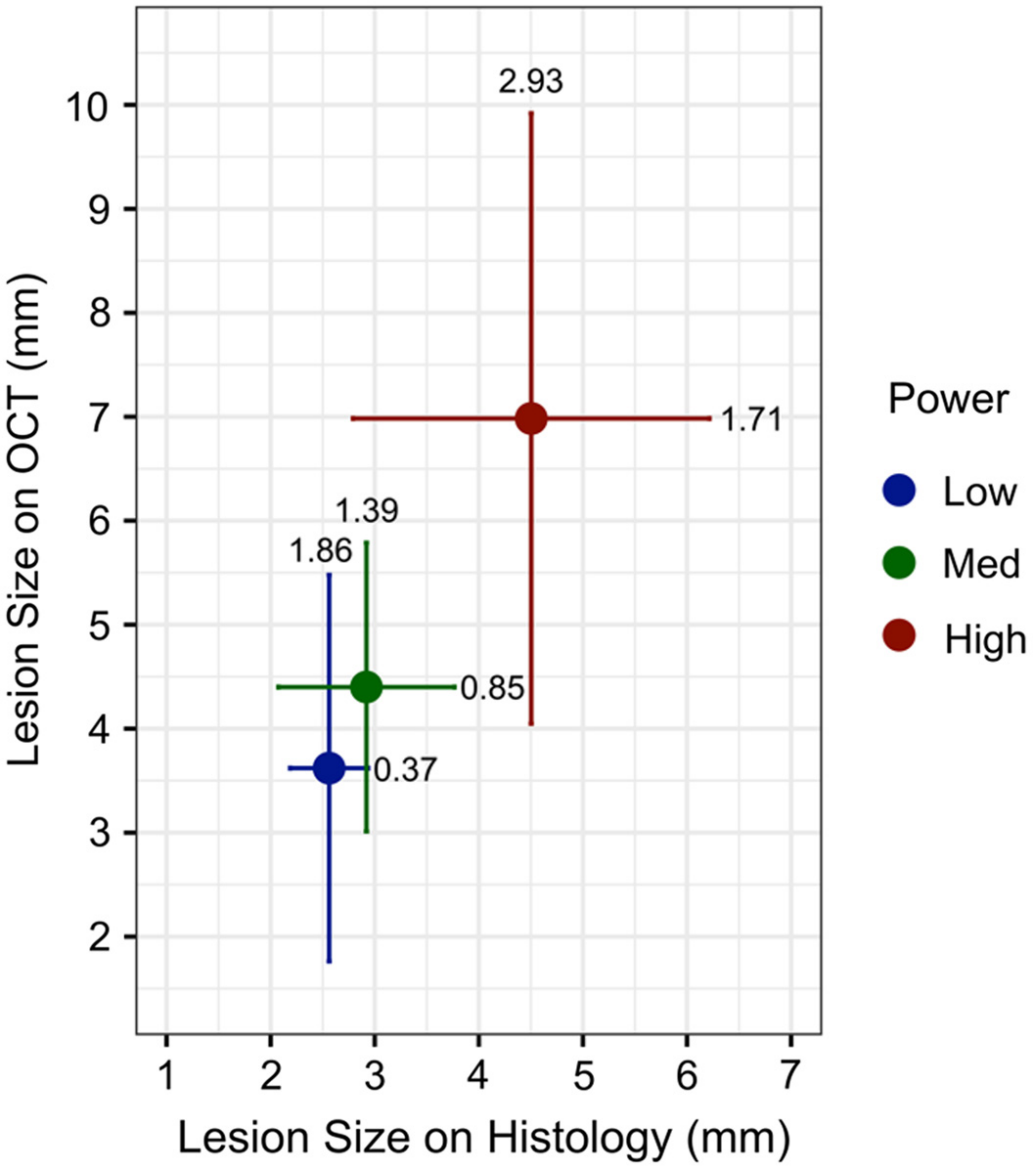
Average lesion size±standard deviation for each lesion as measured with OCT and histology. OCT measurements of lesions treated with high power (36 W) were significantly larger than those treated with low power (16 W) (p=0.038).

Using these criteria, electrothermal injury was identified on OCT images in all but one histology-confirmed lesion (22/23, 95.7%). This medium-power (26-W) lesion measured 4.1 mm on histology and was suspicious for injury on OCT images, but it did not meet the OCT criteria due to its presence on a fold and was therefore considered a false negative. Among the four low-power lesions that did not meet the criteria for injury on histology, three were absent and one false positive was identified as a lesion of 0.9-mm length on OCT images. The average lesion size for lesions that met the criteria for injury on both OCT and histology is shown in Fig. 5. Comparison of OCT to histology measurements revealed a moderate positive correlation (Pearson correlation coefficient, r=0.65, p=0.00087).

### 3.3 Comparison of Power Levels on OCT Images

Morphological features of lesions treated at all power levels on OCT images did not noticeably differ. The extent of damage observed circumferentially (Fig. 4) and measured longitudinally (Table 1) was the only characteristic that correlated with the power level. The lesion size as measured on OCT differed among power groups (p=0.024, ANOVA). Only the difference between low- and high-power lesions as measured on OCT was statistically significant (p=0.038, Tukey’s HSD).

**Table 1:** Comparison of lesion size among power groups as measured with OCT and histology, presented as mean ± standard deviation.

Treatment power	Mean OCT lesion size (mm)	Mean histology lesion size (mm)
Low (n=5)	3.6±1.9	2.6±0.4
Medium (n=8)	4.4±1.4	2.9±0.9
High (n=9)	7.0±2.9	4.5±1.7

Note: n refers to the number of injured ureter segments assessed on both OCT and histology. Measurements for segments that did not meet the criteria for injury on both OCT and histology were excluded. The Pearson correlation coefficient between OCT and histology measurements was statistically significant (r=0.65, p=0.00087).

### 3.4 Physician Detection Study

The results of the physician detection accuracy study are summarized in Table 2. The average time to complete the test was 28±7  min. An average of 112.3/126 (89%) normal and injured images were correctly interpreted by three physicians. The average sensitivity and specificity were 82% and 96%, respectively. Five images with injury consistently received false negative interpretations by all three physicians. An example is shown in Fig. 7. These images demonstrated decreased reflectivity in the lamina propria layer (criteria 1), whereas the microstructural detail in the smooth muscle layer remained unaffected or displayed a slight decrease in contrast (criteria 2).

**Table 2:** Results of the physician lesion detection accuracy study.

Physician	Accuracy	Sensitivity	Specificity	Time to complete (min)
P1	108/126 (86%)	47/63 (75%)	61/63 (97%)	31
P2	115/126 (91%)	54/63 (86%)	61/63 (97%)	20
P3	114/126 (90%)	54/63 (86%)	60/63 (95%)	33
Average	112.3/126 (89%)	51.7/63 (82%)	60.7/63 (96%)	28±7

Note: Following training, three physicians (P1, P2, and P3) classified 126 images as normal or injured with similar time and accuracy.

## 4 Discussion

There is an unmet clinical need for accurate and timely detection of iatrogenic ureteral injury secondary to the use of electrosurgical devices. Herein, we demonstrate an accurate (96%) and measurable detection of electrothermal ureteral injury by an experienced interpreter using a catheter-mounted OCT endoscopy system in the *ex-vivo* setting. Notably, OCT images revealed detectable injury in areas that showed mild to no externally visible signs of injury. While longitudinal outcomes in the live setting were not directly investigated, the amount of energy applied (16 W) is sufficient to induce significant ureteral obstruction and stricture formation.^15^ Our findings underscore the ability of OCT to provide a more thorough and timely evaluation of the spatial and morphological extent of electrothermal ureteral injury. The sub-millimeter diameter scale of OCT endoscopes enables their use as a minimally invasive adjunct to the standard cystoscopy workflow.

In agreement with previous literature, our results demonstrate that OCT can feasibly show the layered organization of normal porcine^19^^,^^20^^,^^22^ and human^21^ ureters. The hyperreflective characteristic features of normal ureters are related to refractive index mismatches and the backreflection of light from organized tissue structures, such as fibrillary collagens and smooth muscle fibers. Electrocoagulation causes protein denaturation and alters the optical properties of tissue, such as scattering, birefringence, and attenuation.^24^^–^^26^ While several studies have demonstrated the potential of OCT to detect alterations related to thermal injury,^16^^,^^27^ we are the first to provide a qualitative description of accurate, standardizable markers of injury in the ureters. In this study, an experienced observer identified injury as areas with decreased contrast and hyporeflectivity on OCT images. Despite one false positive lesion measured, which was attributed to scattering induced by dehydration, these qualitative features were used to provide accurate and agreeable measurements of lesion size in comparison to histology. In the clinical setting, this approach could be used to estimate the severity of injury, inform prognosis, and prompt timely management before the occurrence of serious postoperative complications such as stricture or renal failure.^1^^,^^4^

Despite having no prior OCT experience and only 1 h of training, three physicians in diverse fields differentiated normal from injured OCT images with a high mean detection accuracy (89%). Few false positives (96% mean specificity) were identified, particularly in the presence of attenuating and scattering artifacts that resembled injury. These artifacts occurred most frequently in areas with non-concentric probe alignment, such as tissue folds in larger ureters. In the clinical setting, a centering mechanism such as a balloon or irrigation system is likely needed to mitigate the variability in ureteral diameters.^28^ The low false negative rate (82% sensitivity) shows significant improvement compared with the 38% to 53% intraoperative detection rates for thermal injury^1^ and could minimize reliance on non-specific methods, such as gross inspection or cystoscopy. An important limitation was the comparison of physician interpretation of a single image to labels made by an experienced, unblinded OCT image interpreter with access to additional three-dimensional (3D) volumetric information. In the clinical scenario, physicians could compare suspicious areas to normal and repeat acquisition to better differentiate artifacts from injury.

Despite the small sample size and the subjective nature of this *ex-vivo* study, our results warrant further investigation. In previous work, we developed a convolutional neural network capable of predicting lesion location based on quantifiable alterations in intensity on OCT images.^29^ In future work, we will investigate quantitative measures of injury^24^^–^^26^ to supplement the subjective interpretation of OCT image features. With further development and image feature quantification, automated algorithms could improve the standardization of clinical decision making and potentially reveal microstructural insights or predictors of outcomes not visible to the human eye.^30^^,^^31^ Future work is also needed to demonstrate safety and feasibility in the clinical setting, which is subject to blood and urine flow, obstacles in endoscopic access, and may pose concerns for perforation in areas weakened from injury. In this study, some untreated regions showed isolated epithelial denudation <1  cm in length on histology. Whether this was an artifact of tissue processing and handling or due to damage from the OCT probe, these regions are likely to re-epithelialize *in vivo*.^15^

## 5 Conclusion

Endoscopic OCT was evaluated as a method to detect electrothermal injury in *ex-vivo* porcine ureters. In this study, OCT images provided spatial and morphological information, which enabled accurate, reproducible detection by trained physicians. Our results collectively suggest that endoscopic OCT has the potential to improve and standardize the detection of iatrogenic ureteral injury. Further preclinical investigation is needed to understand its clinical utility and safety.

## 6 Appendix: Supplemental Figures

**Fig. 6:**
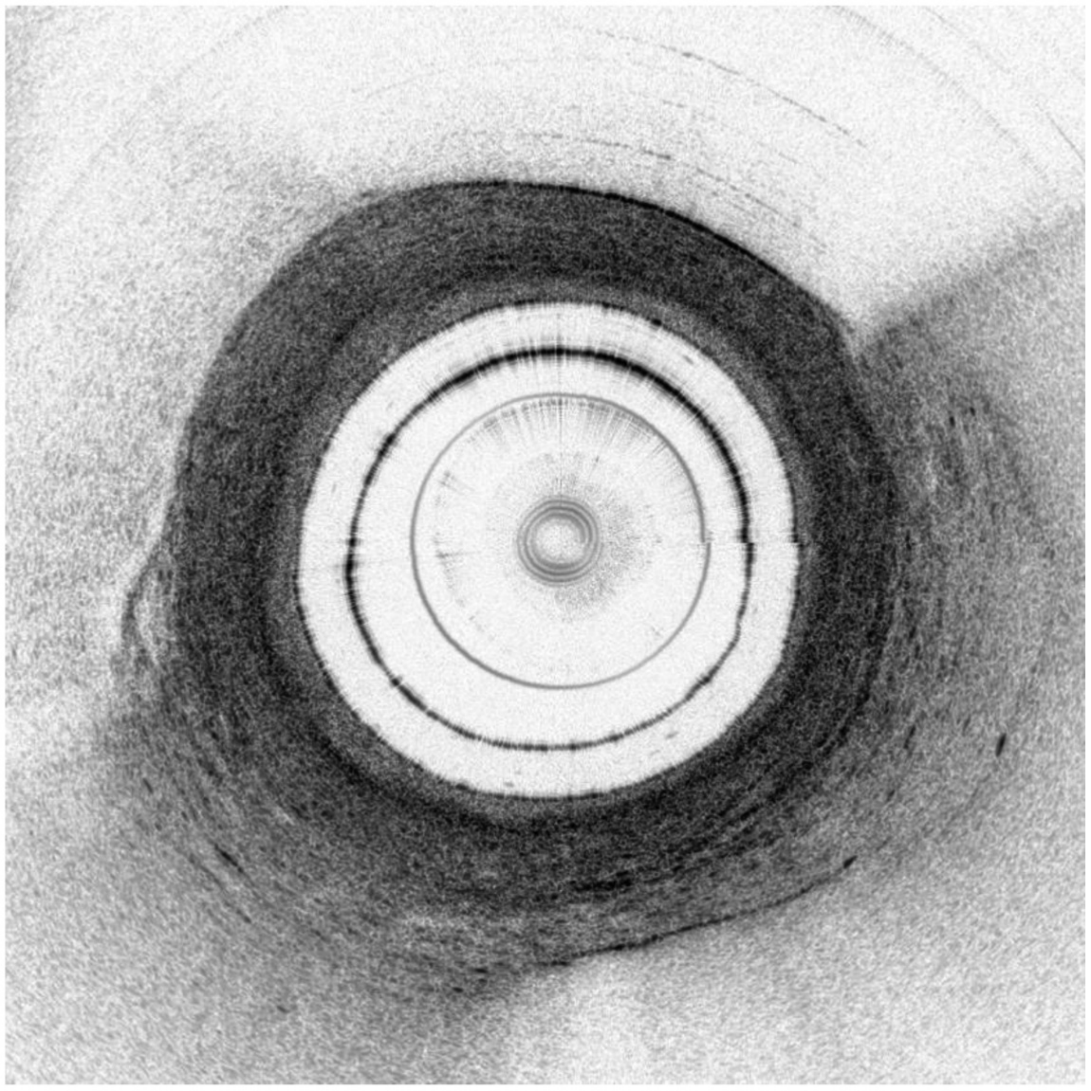
OCT image stack of an explanted porcine ureter, which was externally treated with high-power (36-W) electrothermal energy. The video highlights characteristic features of normal tissue at the beginning and the end and demonstrates both lesion criteria, including lightening of the lamina propria layer and decreased contrast in the smooth muscle layer, as well as an increase in the circumferential extent and severity of injury toward the lesion center (Video 1, MOV, 10.5 MB [URL: https://doi.org/10.1117/1.BIOS.1.1.015001.s1]).

**Fig. 7:**
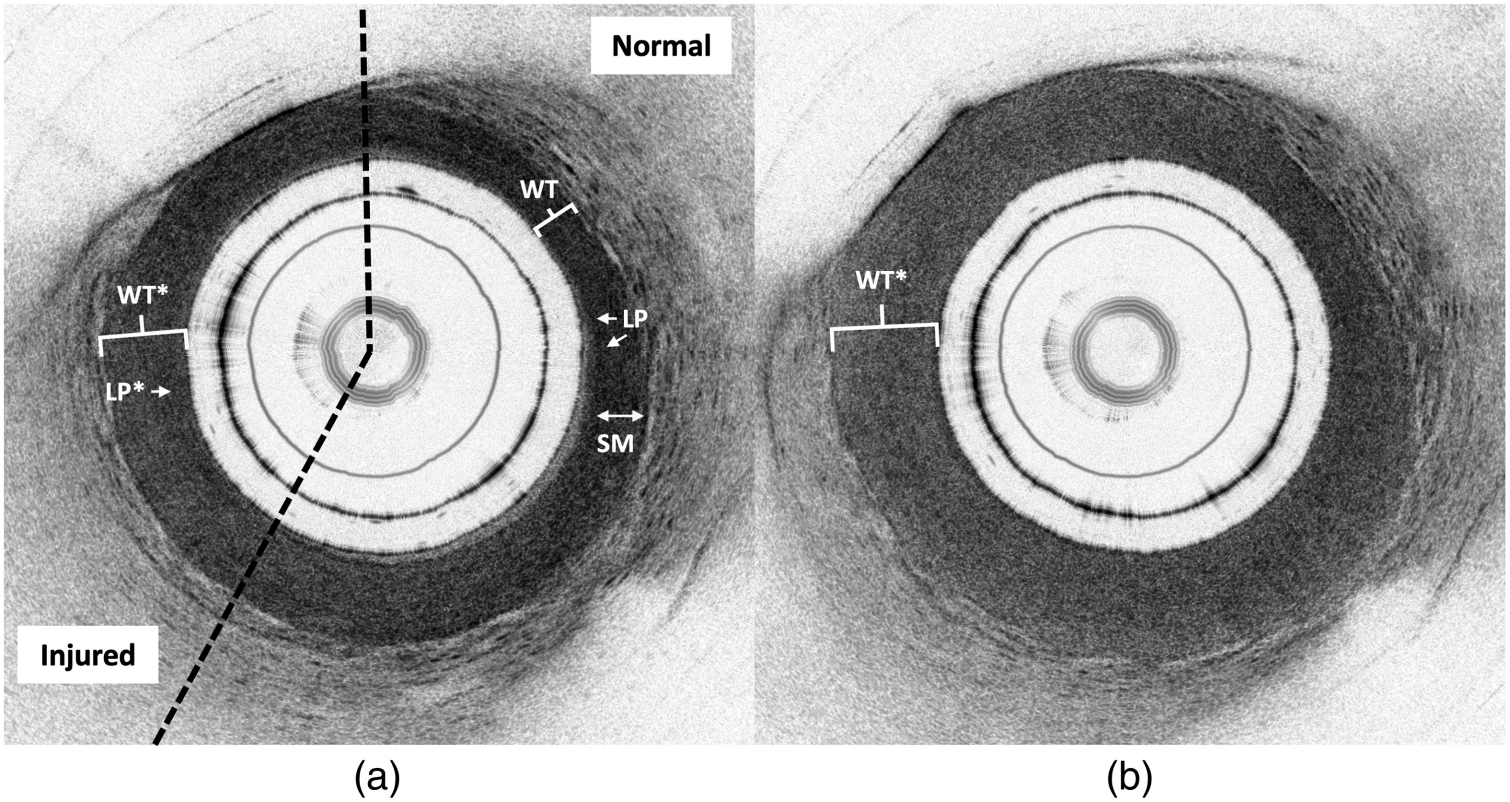
OCT images of one medium power (26 W) lesion, which was incorrectly interpreted by all physicians near the boundary (a) and correctly interpreted by all physicians at the lesion center (b). (a) The injured area showed lightening of the lamina propria (LP*) while contrast and heterogeneity in the smooth muscle layer was intact. Swelling, or increased wall thickness (WT*) compared to normal (WT), was an appreciable feature but was not used as formal criteria for injury. The dotted lines show the approximate boundaries of the lesion, which fulfills only the first injury criteria. Fulfillment of one criterion was sufficient for the detection of injury. (b) Injury was more obvious at the lesion center, which appeared homogeneous, fulfilled both injury criteria, and demonstrated swelling (WT*). This result suggests that with access to additional volumetric information, the physicians would have been able to identify the presence of the lesion.

The following supplemental figures include an OCT image stack of an explanted porcine ureter (Fig. 6) and OCT images of one medium power lesion (Fig. 7).

## Supplementary Material

10.1117/1.BIOS.1.1.015001.s1

## Data Availability

The datasets supporting the conclusions of this article will be made available at the University of Arizona Research Data Repository (ReDATA) at the time of publication (doi: 10.25422/azu.data.25430716).
